# The BET Inhibitor JQ1 Potentiates the Anticlonogenic Effect of Radiation in Pancreatic Cancer Cells

**DOI:** 10.3389/fonc.2022.925718

**Published:** 2022-06-20

**Authors:** Patrick L. Garcia, Aubrey L. Miller, Ling Zeng, Robert C. A. M. van Waardenburg, Eddy S. Yang, Karina J. Yoon

**Affiliations:** ^1^ Department of Pharmacology and Toxicology, University of Alabama at Birmingham, Birmingham, AL, United States; ^2^ Department of Radiation Oncology, University of Alabama at Birmingham, Birmingham, AL, United States; ^3^ UAB Medicine Nursing, Oncology Services, UAB Hospital, Birmingham, AL, United States; ^4^ O’Neal Comprehensive Cancer Center, University of Alabama at Birmingham, Birmingham, AL, United States

**Keywords:** pancreatic cancer, radiation, BET inhibitor, JQ1, DNA damage repair

## Abstract

We reported previously that the BET inhibitor (BETi) JQ1 decreases levels of the DNA repair protein RAD51 and that this decrease is concomitant with increased levels of DNA damage. Based on these findings, we hypothesized that a BETi would augment DNA damage produced by radiation and function as a radiosensitizer. We used clonogenic assays to evaluate the effect of JQ1 ± ionizing radiation (IR) on three pancreatic cancer cell lines *in vitro*. We performed immunofluorescence assays to assess the impact of JQ1 ± IR on DNA damage as reflected by levels of the DNA damage marker γH2AX, and immunoblots to assess levels of the DNA repair protein RAD51. We also compared the effect of these agents on the clonogenic potential of transfectants that expressed contrasting levels of the principle molecular targets of JQ1 (BRD2, BRD4) to determine whether levels of these BET proteins affected sensitivity to JQ1 ± IR. The data show that JQ1 + IR decreased the clonogenic potential of pancreatic cancer cells more than either modality alone. This anticlonogenic effect was associated with increased DNA damage and decreased levels of RAD51. Further, lower levels of BRD2 or BRD4 increased sensitivity to JQ1 and JQ1 + IR, suggesting that pre-treatment levels of BRD2 or BRD4 may predict sensitivity to a BETi or to a BETi + IR. We suggest that a BETi + IR merits evaluation as therapy prior to surgery for pancreatic cancer patients with borderline resectable disease.

## Introduction

Pancreatic cancer (PC) is estimated to become the second leading cause of cancer related deaths in the United States in the next decade (1). The 5-year survival for patients with PC is ~10% (2). Surgical resection is the only potentially curative treatment, but only ~20% of patients are eligible for resection at diagnosis (3, 4). Criteria that determine eligibility for surgical resection have included absence of metastatic lesions and limited invasion into arteries of the mesenteric-portal axis (5). Several approaches to increase the number of eligible cases for resection have been evaluated for patients with pancreatic ductal adenocarcinoma (PDAC), the most common form of PC. For example, combinations of a radiosensitizing agent and radiotherapy increases the number of patients with nonmetastatic disease that are eligible for resection, based on assessments that negative margin resection is possible post treatment (6–8). Two noteworthy studies evaluated the benefits of combining radiation with 5-fluorouracil (5-FU) or with FOLFIRINOX (5-FU, oxaliplatin, irinotecan, and leucovorin). The first, a prospective study, addressed the benefit of preoperative 5-FU + IR (50.4-56 gy) in borderline resectable PC. The study was conducted with 15 patients in 2001 by Mehta and colleagues (9). This approach converted 9/15 (60%) patients from surgically ineligible to eligible. The second, a retrospective study in 2014 by Christians, et al. analyzed the potential benefit for PC patients of FOLFIRINOX followed by gemcitabine or capecitabine and radiation (50.4 gy) (10). Twelve out of eighteen patients met the criteria for resection post therapy and had subsequent margin negative resections. Of these 12 patients, 7 were alive at termination of study 35 months post resection, and 5 of the 7 had no evidence of disease 18-35 months after diagnosis. Our study addresses whether the BETi JQ1, which we have shown to have anti-tumor efficacy as a single agent in preclinical models of PDAC, is also a radiosensitizer.

JQ1 inhibits the activity of BET bromodomain proteins (BRD2/3/4) by competitively inhibiting the association of BET proteins with acetylated lysine residues of binding partners (11). BRD2 and BRD4 are the main targets of BET inhibitors (12). The most well characterized effect of BET inhibitors is a decrease in binding of BET-dependent transcriptional complexes to promoter and enhancer regions of specific genes (13, 14). This inhibition, in turn, decreases expression of genes dependent on this mechanism of transcription. Recent literature suggests BET inhibitors may also decrease DNA repair mechanisms that depend on BRD4 activity (15–17). Consistent with this finding, we have shown that JQ1 increases levels of the DNA damage marker γH2AX and decreases expression of the NHEJ DNA repair protein Ku80 and the HR DNA repair protein RAD51 in models of PDAC of human origin (18).

Work presented here addresses whether JQ1 functions as a radiosensitizer in PC models *in vitro* and whether inhibition of BET protein activity or downregulation of BRD2 or BRD4 expression contributes to the potency of this combination. The work is based on the hypothesis that JQ1-mediated decreases in BET activity and consequent DNA repair deficiency sensitize PC cells to ionizing radiation. The long-range goal of the work is to determine if BET inhibition + IR comprises an effective pre-surgical treatment for patients that present with borderline resectable PDAC.

## Materials and Methods

### Cell Culture and Chemicals

Panc1, BxPC3, and MiaPaCa2 PC cell lines were purchased from the American Type Culture Collection (ATCC, Manassas, VA, USA). Cells were cultured in DMEM (Fisher Scientific, Waltham, MA, USA) containing 10% fetal bovine serum (Atlanta Biologicals, Flowery Branch, GA, USA) and 2 mM L-glutamine (Fisher Scientific). Each PC cell line was tested for mycoplasma using MycoAlert™ Plus Detection Kit (Lonza, Walkersville, MD, USA) and were negative. JQ1 (HY-13030, MedChem Express, Monmouth Junction, NJ, USA) stock solutions were prepared in DMSO, and diluted to the appropriate concentration in complete DMEM media. Vehicle control was DMSO (<0.01%).

### Clonogenic Assay

PC cells were plated in 24-well plates (Panc1: 250 cells/well, BxPC3: 400 cells/well, and MiaPaCa2: 200 cells/well) and allowed to adhere for 24h. After being exposed to JQ1 for 72h or 120h and then irradiated (IR), cells were propagated in drug-free media for the equivalent of 5-6 cell doubling times (doubling times: Panc1, 48 hours; BxPC3, 52 hours; MiaPaCa2, 30 hours.). Cells were stained with crystal violet (0.025%) at 10, 14 and 7 days after exposure to drug. Quantification was performed by counting stained colonies of >50 cells, using an Olympus CK2 microscope (Olympus Scientific Solutions Americas Corp., Waltham, MA, USA). The plating efficiency (PE) was calculated as: PE = (number of colonies counted)/(number of cells plated). The clonogenic survival fraction (SF) for each treatment was calculated as: SF = [(# of colonies counted)/(# cells plated x PE) x 100] (15, 19–21). Data are reported as average survival fraction ± SEM.

### Immunoblot

Cells were plated (3 x10^5^ cells/well) in 6-well plates and allowed to adhere for 24h, exposed to JQ1 for 48h, and then irradiated (IR). Cells were harvested 1h or 24h post IR. Whole cell lysates were prepared in NP-40 containing protease inhibitors (Fisher Scientific). Primary antibodies used were: Cleaved-PARP (5625, Cell Signaling, Denvers, MA, 1:1,000), γH2AX (9718S, Cell Signaling, 1:1,000), RAD51 (ab88572, Abcam, Waltham, MA, 1:1,000), GAPDH (97166, Cell Signaling, 1:10,000), BRD2 (5848, Cell Signaling, 1:1,000) and BRD4 (13440, Cell Signaling, 1:1,000). Secondary antibodies used were: HRP goat anti-rabbit IgG (6721, Abcam, 1:50,000) and HRP anti-mouse IgG (7076, Cell Signaling, 1:5,000). Immunoblots were quantitated using ImageJ software. Data were normalized to respective loading controls and then to the DMSO control.

### Immunofluorescence

BxPC3 cells were seeded (1 x 10^4^ cells/well) on chamber slides (Lab-Tek^®^, Rochester, NY, USA) and incubated under standard conditions for 24h prior to drug or IR exposure. Cells were then exposed to JQ1 for 48h + 0, 4, or 8 gy IR and resuspended in drug-free medium for 1 or 24 hours, as indicated. At harvest, cells were washed with PBS, fixed with 10% neutral buffered formalin (NBF) (Fisher Scientific), and permeabilized with 0.1% Triton x-100 (Fisher Scientific). Blocking of non-specific binding was performed in 2% bovine serum albumin (BSA) (Fisher Scientific). γH2AX foci (05-636, Millipore Sigma, Burlington, MA, 1:500) were detected using AlexFluor 488 nm donkey anti-rabbit conjugated secondary antibody (1:500), and nuclei were stained with DAPI (1:20,000). Images were taken using a Zeiss Observer Z.1 microscope and photomicrographs were processed with Zen 2011 Blue imaging software (Zeiss, Dublin, CA, USA).

### Generation of Stable Transfectants

The protocol used to generate stable transfectants of Panc1 cells that express contrasting levels of BRD2 or BRD4 is published (18). Briefly, Panc1 cells were transfected using PEI (Polysciences Inc., Warrington, VA, USA) and Mission shRNA (Millipore Sigma) targeting BRD2 or BRD4. shGFP served as vector control (Addgene, Watertown, MA, USA). Selection of stable transfectants was carried out using 10 μg/ml puromycin (ENZO Life Sciences, Farmingdale, NY, USA).

### Irradiation

Radiation experiments were carried out using a Kimtron IC-320 Series Biological Irradiator (Kimtron Medical Inc., Oxford, CT, USA). Cells were exposed to 4 or 8 gy IR using a 320 kV x-ray tube with a maximum output of 3200 watts, with parameters set on the SC-500 Series II controller (Kimtron Medical Inc.). In addition, the IR dose was also measured by a PTW Unidose E dosimeter (PTW, Freiburg, Germany).

### Statistical Analysis

Statistical analysis was performed using GraphPad Prism 8.0 and 9.0 (San Diego, CA, USA). Quantitation of clonogenic assays, IF assays for BxPC3 cells and IB for Panc1, MiaPaCa2 cells was done using one-way ANOVA followed by Tukey multiple comparisons. P<0.05 was considered significant. All experiments were done a minimum of three times.

## Results

### JQ1 + IR Reduced Clonogenic Potential More Than JQ1 or IR as a Single Modality

**Figure 1:**
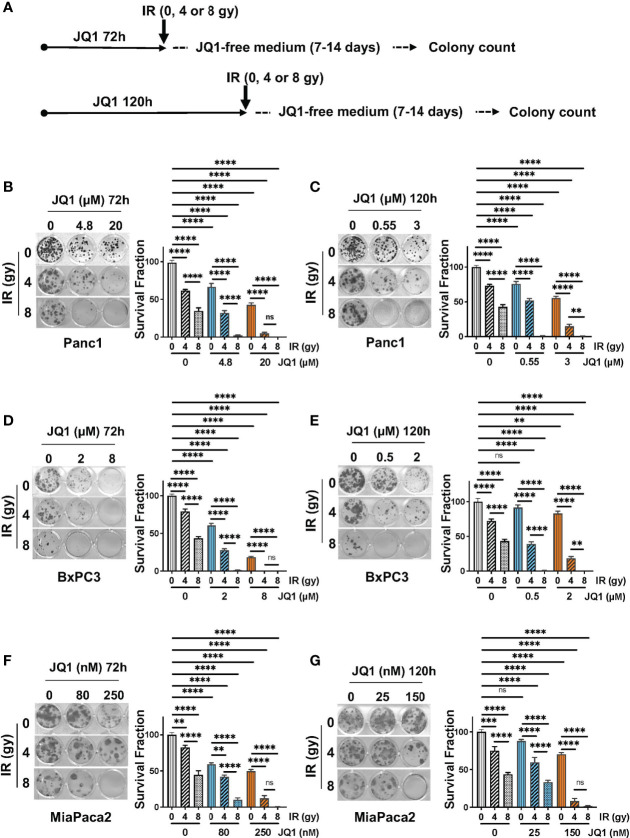
The combination of JQ1 + IR reduces clonogenic potential more than JQ1 or IR as a single modality. **(A)** Schematic of the clonogenic assay protocol. Cells were exposed to JQ1 for 72h and 120h. **(B, C)** Images of representative plates showing colonies produced by Panc1 cells at **(B)** 72 hours or **(C)** 120 hours exposure to JQ1 ± IR. **(D, E)** Images of representative plates showing colonies (left panel) and survival fraction (right panel) of BxPC3 cells at **(D)** 72 hours or **(E)** 120 hours after treatment. **(F, G)** Images of representative plates showing colonies (left panel) and survival fraction (right panel) of MiaPaCa2 cells at **(F)** 72 hours or **(G)** 120 hours after treatment. Data are reported as average survival fraction ± SEM, as shown on bar graphs to the right of each plate image. One-way ANOVA followed by Tukey multi comparison analysis was performed as described in Materials and Methods. **p < 0.01, ***p < 0.001, ****p < 0.0001. ns, not significant.

Clonogenic assays reflect cell reproductive capacity and are used as *in vitro* assessments of efficacy of IR and chemotherapeutic agents (20). We performed clonogenic assays using three PC cell lines to evaluate whether the combination of JQ1 + IR was more effective than either modality alone (**Figure 1**). The two concentrations of JQ1 used for each cell line reflect the approximate IC25 and IC50 for that cell line at two different exposure times. The two exposure times, 72h or 120h, were used to compare the impact of JQ1 at a higher dose for a shorter time with a lower dose for a longer time (**Figure 1A**). We used two doses of IR, 4 or 8 gy, to determine if increasing the dose provided added benefit when combined with a lower vs higher dose of JQ1. All concentrations used for JQ1 are achievable in plasma of murine models at nontoxic doses (22). Immediately after IR, cells were placed in drug-free media and propagated for an additional 5-6 cell doubling times (7-14 days, depending on the cell line).

*Panc1 cells.* Cells that were not exposed to JQ1 or IR (control) produced 40 ± 1 colonies. IR alone, 4 or 8 gy, reduced clonogenic survival fraction by 37% and 64%, respectively, compared to control (no JQ1 or IR). Exposure to JQ1 alone produced a dose-dependent reduction in colony number of ~32-56% compared to controls (p<0.0001) (**Figure 1B**). The combination of JQ1 + IR at all doses used was more effective than either modality alone. Notably, cells exposed to 8gy IR + 20 µM JQ1 for 72h or to 8 gy IR + 0.55 or 3 µM JQ1 for 120h completely suppressed colony formation. (**Figures 1B, C**). The data demonstrate that, in combination with each dose and time of JQ1 evaluated, 8 gy IR provided greater inhibition of clonogenic potential *in vitro* than 4 gy.

*BxPC3 cells.* Control BxPC3 cells produced 22 ± 1 colonies. 4 or 8 gy IR reduced colony number by 21% or 57%, respectively (**Figure 1D**). JQ1 alone (2 or 8 µM) decreased survival fraction by 39-82%, respectively. Similar to Panc1 cells, the combination of JQ1 + IR was more effective than either agent alone: BxPC3 survival fraction was completely inhibited when exposed to 4 gy IR + 8 µM JQ1 or 8 gy IR + 2 µM or 8 gy IR + 8 µM JQ1 at 72h (**Figure 1D**). A similar additive effect was seen in cells exposed to the lower doses of JQ1 for 120h (**Figure 1E**).

*MiaPaca2 cells.* Control MiaPaCa2 cells produced 24 ± 1 colonies. Radiation (4 or 8 gy) as a single treatment reduced survival fraction by 18%, or 56%, respectively (**Figure 1F**). JQ1 (80 or 250 nM) as a single modality decreased colony number by 41%, 50%, respectively. The combination decreased survival fraction by 58-99% compared to control (**Figure 1F**). As seen with Panc1 and BxPC3 cells, survival fraction was reduced to 99% following exposure to 8 gy IR + 250 nM JQ1 for 72h. Similar results were observed with cells exposed to JQ1 for 120h (**Figure 1G**).

Taken together, the data show that a higher dose of JQ1 x shorter exposure (72h) + IR and a lower dose of JQ1 x longer exposure (120h) + IR had similar impact on colony numbers. Further, concentrations of the BETi JQ1 achievable in murine plasma (up to 24 µM) at nontoxic doses can be effectively combined with IR to decrease the clonogenic potential of PC cells (22).

### Immunofluorescence Data Demonstrate That JQ1 + IR Increased Levels of the DNA Damage Marker γH2AX and Decreased Levels of the DNA Repair Protein RAD51 in BxPC3 Cells

BET-associated transcription complexes regulate expression of many genes. We showed previously that JQ1 decreased expression of DNA repair proteins Ku80 and RAD51 in *in vivo* PDAC models (18). We also reported that JQ1 increased levels of DNA damage, as reflected by an increase in levels of the damage marker γH2AX (18, 23). These observations suggested that JQ1 interferes with DNA damage repair. We performed immunofluorescence (IF) and immunoblot (IB) assays to determine whether IR enhanced these two known effects of the BETi JQ1 in BxPC3 PC cells. Doses of JQ1, 2 or 8 µM represent approximate IC25 and IC50 concentrations for this cell line. The exposure time of 48h was based on laboratory experience indicating that increases in γH2AX are readily detectable 48h after exposure to JQ1.

**Figure 2:**
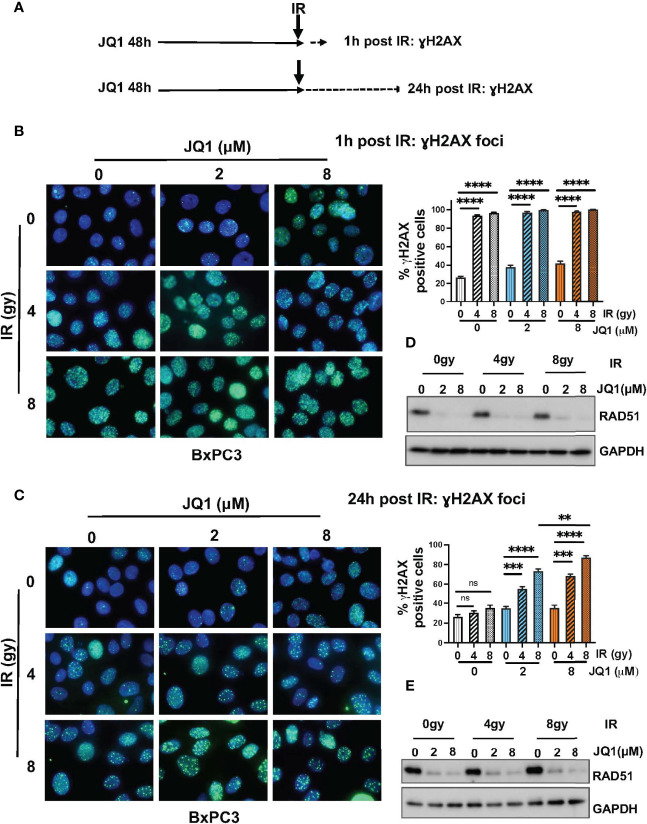
Immunofluorescence and immunoblot data show that JQ1 + IR increases levels of the DNA damage marker γH2AX and decreases levels of the DNA repair protein RAD51 in BxPC3 cells. The combination is more effective than either single modality. **(A)** Schematic of exposure and harvest times of cells exposed to JQ1 and IR prior to evaluating levels of γH2AX and RAD51. **(B, C)** Images of representative immunofluorescence (IF) data at **(B)** 1 hour post IR or **(C)** 24h post IR. Quantitation of γH2AX foci is presented on bar graphs to the right of the IF images. **(D, E)** Representative immunoblots for RAD51 at **(D)** 1 hour and **(E)** 24 hours post IR. One-Way ANOVA followed by Tukey multi comparison analysis was performed as described in Materials and Methods. **p < 0.01, ***p < 0.001, ****p < 0.0001. ns, not significant.

Cells were exposed to JQ1 for 48h and irradiated. Analyses were performed at 1h and at 24h post IR (**Figure 2A**). We used IF to evaluate formation of γH2AX foci as a measure of DNA damage in BxPC3 cells exposed to JQ1 (2 or 8 µM) ± IR (4 or 8 gy) (**Figures 2B, C**). Cells with >5 foci were designated γH2AX positive. We used IB to assess the effect of JQ1 ± IR on expression of the DNA damage repair protein RAD51 (**Figures 2D, E**). At 1h post IR, the data show that: JQ1 increased γH2AX foci and decreased RAD51 levels compared to controls; that 4 gy IR augmented the effect of JQ1 on γH2AX; and that the effect of JQ1 + 4 gy IR was equivalent to that of JQ1 + 8 gy IR. In contrast, the effect of 4 vs 8 gy IR at 24h post irradiation (compare **Figures 2B, C**) at 2 or 8 µM JQ1, 8 gy IR increased γH2AX foci more than JQ1 + 4 gy IR (p<0.01). As anticipated, IR alone had little effect on the level of detectable RAD51 protein. The data suggest that JQ1 inhibits DNA repair and acts as a radiosensitizer and that the degree of sensitization is dose-dependent.

### Immunoblot Data Demonstrate That JQ1 + IR Increased Levels of the DNA Damage Marker γH2AX and Decreased Levels of the DNA Repair Protein RAD51 in Panc1 and MiaPaCa2 Pancreatic Cancer Cells, and That the Increase in γH2AX Was Concomitant With an Increase in the Apoptosis Marker Cleaved PARP

**Figure 3:**
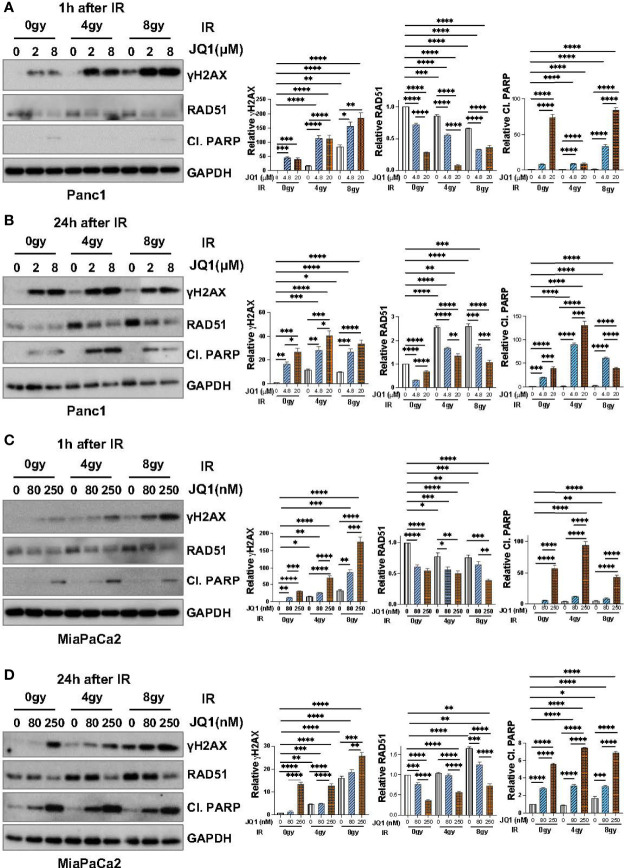
Immunoblot data demonstrate that JQ1 + IR increases levels of the DNA damage marker γH2AX and decreases levels of the DNA repair protein RAD51 in Panc1 and MiaPaCa2 pancreatic cancer cells, and that the increase in γH2AX is concomitant with an increase in the apoptosis marker cleaved PARP (Cl. PARP). Images of representative immunoblots of Panc1 cells exposed to JQ1 ± IR, 1h after IR **(A)** or 24h after IR **(B)**, and of MiaPaCa2 cells 1h after IR **(C)** or 24h after IR **(D)**. Quantitation of γH2AX, RAD51 or Cl. PARP, was done using ImageJ software and analyzed using one-way ANOVA followed by Tukey multi comparison analysis. Quantitation is shown to the right of each immunoblot images as a bar graphs. *p < 005, **p < 0.01, ***p < 0.001, ****p < 0.0001.

To confirm that data seen with BxPC3 cells was not unique to that PC cell line, we used IB to examine the effect of JQ1 ± IR on γH2AX and RAD51 levels in Panc1 and MiaPaCa2 cells (**Figure 3**). We also evaluated levels of cleaved PARP (Cl. PARP) to determine if this marker of apoptosis increased in parallel with an increase in γH2AX. We hypothesized that if the observed increase in DNA damage affected cell viability, Cl. PARP would increase simultaneously with γH2AX.

As was seen with BxPC3 cells, IR or JQ1 as single agents increased the level of γH2AX in Panc1 cells (**Figures 3A, B**) and JQ1 + IR increased the levels of γH2AX more than JQ1 or IR as a single modality. Also similar to data with BxPC3 cells, JQ1 decreased RAD51 levels (p<0.0001) at both time points post IR and IR had little if any effect on RAD51.

As with BxPC3 and Panc1 cells, in MiaPaCa2 cells JQ1 + IR increased γH2AX compared to JQ1 or IR alone (p<0.0001). Additionally, similar to Panc1 cells, JQ1 + IR increased levels of Cl. PARP at 24h, concomitant with the increase in γH2AX (p<0.0001) (**Figures 3C, D**). The data show that JQ1 and JQ1 + IR increase the levels of markers that reflect DNA damage and apoptosis. The data demonstrate simultaneous increases in DNA damage and apoptosis in cells exposed to JQ1 + IR greater than JQ1 or IR, and suggest that the BETi JQ1 functions as a radiosensitizer.

### Downregulation of BRD2 and BRD4 Enhanced Sensitivity to JQ1 + IR

**Figure 4:**
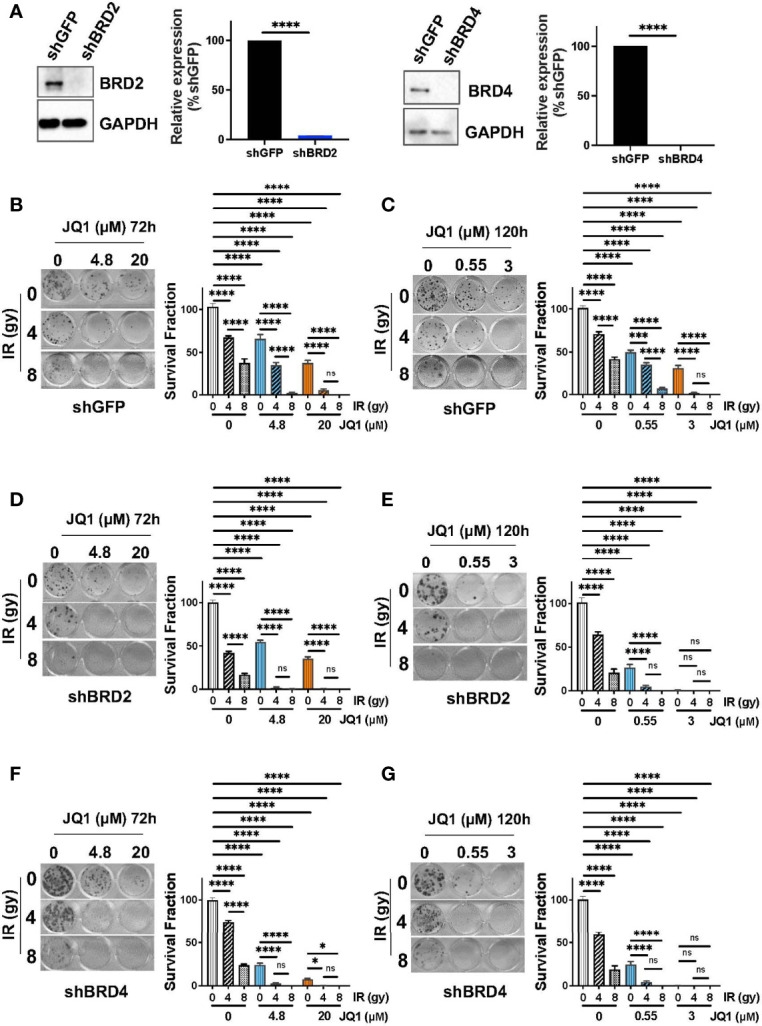
Downregulation of BRD2 or BRD4 enhances sensitivity to JQ1 + IR. **(A)** Levels of BRD2 and BRD4 protein were lower in Panc1 cells transfected with shBRD2 or shBRD4 than in shGFP control transfectants. Quantitation of expression level was done as in Materials and Methods. ****p<0.0001. **(B, C)** Images of representative clonogenic assays showing colonies for shGFP transfectants exposed to JQ1 for **(B)** 72 hours or **(C)** 120 hours ± IR or for **(D, E)** shBRD2 transfectants or for **(F, G)** shBRD4 transfectants. Average survival fraction ± SEM is shown to the right of each image as a bar graph. Quantitation was done as in Materials and Methods. *p < 005, ***p < 0.001, ****p < 0.0001. ns, not significant.

We next addressed a mechanistic aspect of JQ1 + IR on PC cells. BRD2 and BRD4 are the principal molecular targets of JQ1 (12). We considered the possibilities that lower levels of BRD2 or BRD4 would decrease JQ1 potency due to a relative lack of target protein(s) or, alternatively, lower levels of BRD2 or BRD4 would increase JQ1 potency because lower levels of drug might be needed to inhibit a greater percentage of BET protein activity. We transfected Panc1 cells with shBRD2 or shBRD4 to decrease expression of each protein by 96% or 99%, respectively, (**Figure 4A**) and evaluated the sensitivity of these and of shGFP control transfectants to JQ1 ± IR using clonogenic assays. As for experiments in **Figures 1B, C**, we used two concentrations of JQ1 and two exposure times: 4.8 or 20 µM for 72h (**Figures 4B, D, F**) and 0.55 and 3 µM for 120h (**Figures 4C, E, G**) ± 4 or 8 gy IR. Immediately after irradiation, cells were placed in drug-free media and propagated for an additional 10 days.

In all three transfectants, JQ1 or IR as a single modality reduced colony number compared to controls (p<0.0001) and the combination reduced survival fraction more than either modality alone (p<0.0001). The data show that 4.8 or 20 µM JQ1 for 72h + 4 gy or 8 gy IR abrogated colonies in shBRD2 transfectants and reduced survival fraction by 97-100% in shBRD4 transfectants, compared to controls (**Figures 4B, D, F**). Also, 0.55 or 3 μM JQ1 x 120h abrogated colony formation in shBRD2 and shBRD4 transfectants (**Figures 4C, E, G**). PC cells with lower levels of BRD2 or BRD4 were more sensitive to JQ1 as a single agent and to JQ1 + IR. Notably, the combination abrogated clonogenic potential at concentrations of JQ1 that are achievable in murine plasma at nontoxic doses, in a dose- and time-dependent manner.

Taken together, our data suggest that the levels of BRD2 or BRD4 may represent a marker of sensitivity to BET inhibitors, and that a BET inhibitor can be effectively combined with IR to inhibit PC cell proliferation.

## Discussion

This study evaluated whether pharmacologic inhibition of BET bromodomain activity by the BETi JQ1 or downregulation of its molecular targets BRD2 or BRD4 potentiated the anti-clonogenic effect of IR *in vitro*. The data show that JQ1 + IR had greater effect than either treatment alone. The anticlonogenic effect of JQ1 + IR was associated with increases in DNA damage and decreases in expression of the DNA repair protein RAD51. Our data suggest that a combination of BET inhibitor and radiation may be a useful strategy to reduce tumor proliferation and further as a neoadjuvant therapy to reduce tumor volume in patients with PC.

BET proteins contribute to multiple cellular processes in normal and tumor cells. The most well characterized function of BET proteins is to facilitate binding of BET-dependent transcription complexes to specific chromatin associated histones, to regulate transcription of target genes (24, 25). Preclinical studies demonstrate a potential utility BET inhibitors for treatment of a variety of solid malignancies including breast, lung, ovarian, and pancreatic cancers (26–30). Recent studies have sought to identify combination strategies that include BET inhibitors, to improve the efficacy of current standards of care. One such study by Karakashev et al. (2017) determined that the BETi JQ1 + the PARPi olaparib inhibited BRCA-proficient ovarian cancer cell proliferation *in vitro* and *in vivo* (16). The authors of that study conclude that the synergy observed with JQ1 + olaparib was associated with a JQ1-mediated decrease in expression of the G2 checkpoint kinase WEE1 and the DNA damage response protein TOPBP1. In 2019, our lab demonstrated the anti-tumor efficacy of this combination in pancreatic patient-derived xenograft (PDX) models (18). Mechanistic studies that were a component of that work showed that RNAi targeting either of the two molecular targets of JQ1, BRD2 or BRD4, decreased levels of the NHEJ repair protein Ku80 and the HR repair protein RAD51. Consistent with that finding, tumors of mice treated with JQ1 + olaparib had higher levels of DNA damage than tumors from mice treated with either drug as a single agent, as reflected by levels of the DNA damage marker γH2AX. The current study focused on RAD51 levels, since RAD51 is considered a representative marker for DNA repair proficiency and is frequently overexpressed in human PDAC tumors (31–34).

Recent studies indicate that this family of proteins also contributes directly to DNA damage repair. Li et al. (2018) demonstrated that the BET protein BRD4 binds to acetylated histones following IR-induced DNA damage and associates with the NHEJ DNA repair protein Ku80 to increase DNA repair in prostate cancer models (35). In that study, JQ1 alone did not increase levels of γH2AX. The latter observation contrasts with our observation in PC cells in that JQ1 as a single agent increases levels of γH2AX. A study by Cameros et al. (2020) showed that the BET inhibitor OTX015 + IR was more effective than OTX015 or IR alone, as reflected by increases in markers of apoptosis and of DNA damage *in vitro* in rhabomyosarcoma cells (36). In that study, increased DNA damage was associated with decreased levels of DNA damage repair proteins RAD51, ATM, and DNA-PK. Mechanistically, Wang et al. (2017) showed that JQ1 inhibits repair of DNA double-strand breaks induced by IR and promotes apoptosis in non-small cell lung cancer cell lines (37). Further, Yang et al. (2017) showed that JQ1 decreased HR DNA repair activity following 10 gy IR in 3 ovarian cancer cell lines (17). Similar to our study, these investigators observed an increase in γH2AX foci and decreased expression of the DSB repair protein RAD51. In that study, ChIP data showed that JQ1 inhibited binding of BRD2/3/4 to the RAD51 promoter, directly repressing expression of RAD51. Our study and published work using multiple types of tumor cell models indicate that JQ1 disrupts DNA damage repair (15–18, 38–40). Our data suggest that JQ1 may function as a radiosensitizer in pancreatic cancer, the first study to address this question. The data also suggest that a BET inhibitor + IR warrants further investigation to determine if this combination increases the number of PC patients eligible for resection.

Approximately 40% of patients diagnosed with PDAC have locally advanced disease at the time of diagnosis (41). For patients with borderline resectable tumors, neoadjuvant therapy of chemotherapy + IR has the potential to facilitate margin free resection and improve prognosis. Recently The National Cancer Research Institute Clinical and Translational Radiotherapy (CTRad) working group released consensus guidelines to encourage clinical trials conducted with novel compounds in conjunction with radiotherapy (42). Our study suggests that combining a BET inhibitor with IR may be a useful strategy to augment the anti-tumor efficacy of IR prior to resection in this patient population. Future work includes evaluation of BRD2 or BRD4 as a marker of sensitivity to JQ1 + IR and comparison of a BET inhibitor ± IR in low vs high level BRD2 or BRD4 expressing preclinical models of PC.

## Data Availability Statement

The original contributions presented in the study are included in the article. Further inquiries can be directed to the corresponding author.

## Author Contributions

Conception and/or design: RvW, EY, KY. Acquisition of data: PG, AM, LZ. Data analysis and interpretation: PG, AM, EY, RvW, KY. Resources: EY, RvW, KY. Writing:-original draft preparation: PG, AM, KY. Writing-review and editing: PG, AM, LZ, EY, RvW, KY. Study supervision: EY, KY. All authors have read and agreed to the final version of the manuscript. All authors contributed to the article and approved the submitted version.

## Funding

This work was supported by the National Institutes of Health (National Cancer Institute) grant R01CA208272 (KY). EY received grant support from Eli Lilly and PUMA Biotechnologies, Inc. that is unrelated to this study. The funders were not involved in the study design, collection, analysis, interpretation of data, the writing of this article or the decision to submit it for publication.

## Conflict of Interest

EY serves as an advisory consultant for AstraZeneca, Bayer, Clovis, and Strata Oncology.

The remaining authors declare that the research was conducted in the absence of any commercial or financial relationships that could be construed as a potential conflict of interest.

## Publisher’s Note

All claims expressed in this article are solely those of the authors and do not necessarily represent those of their affiliated organizations, or those of the publisher, the editors and the reviewers. Any product that may be evaluated in this article, or claim that may be made by its manufacturer, is not guaranteed or endorsed by the publisher.

## References

[1] Rahib L Wehner MR Matrisian LM Nead KT. Estimated Projection of US Cancer Incidence and Death to 2040. JAMA Netw Open (2021) 4(4):e214708. doi: 10.1001/jamanetworkopen.2021.4708 33825840 PMC8027914

[2] Mizrahi JD Surana R Valle JW Shroff RT. Pancreatic Cancer. Lancet (2020) 395(10242):2008–20. doi: 10.1016/S0140-6736(20)30974-0 32593337

[3] Bilimoria KY Bentrem DJ Ko CY Stewart AK Winchester DP Talamonti MS. National Failure to Operate on Early Stage Pancreatic Cancer. Ann Surg (2007) 246(2):173–80. doi: 10.1097/SLA.0b013e3180691579 PMC1933550 17667493

[4] Vincent A Herman J Schulick R Hruban RH Goggins M. Pancreatic Cancer. Lancet (2011) 378(9791):607–20. doi: 10.1016/S0140-6736(10)62307-0 PMC3062508 21620466

[5] Grossberg AJ Chu LC Deig CR Fishman EK Hwang WL Maitra A. Multidisciplinary Standards of Care and Recent Progress in Pancreatic Ductal Adenocarcinoma. CA Cancer J Clin (2020) 70(5):375–403. doi: 10.3322/caac.21626 32683683 PMC7722002

[6] Versteijne E Suker M Groothuis K Akkermans-Vogelaar JM Besselink MG Bonsing BA. Preoperative Chemoradiotherapy Versus Immediate Surgery for Resectable and Borderline Resectable Pancreatic Cancer: Results of the Dutch Randomized Phase III PREOPANC Trial. J Clin Oncol (2020) 38(16):1763–73. doi: 10.1200/JCO.19.02274 PMC8265386 32105518

[7] Park W Chawla A O'Reilly EM. Pancreatic Cancer: A Review. JAMA (2021) 326(9):851–62. doi: 10.1001/jama.2021.13027 PMC9363152 34547082

[8] Vauthey JN Dixon E. AHPBA/SSO/SSAT Consensus Conference on Resectable and Borderline Resectable Pancreatic Cancer: Rationale and Overview of the Conference. Ann Surg Oncol (2009) 16(7):1725–6. doi: 10.1245/s10434-009-0409-5 19396495

[9] Mehta VK Fisher G Ford JA Poen JC Vierra MA Oberhelman H. Preoperative Chemoradiation for Marginally Resectable Adenocarcinoma of the Pancreas. J Gastrointest Surg (2001) 5(1):27–35. doi: 10.1016/S1091-255X(01)80010-X 11309645

[10] Christians KK Tsai S Mahmoud A Ritch P Thomas JP Wiebe L. Neoadjuvant FOLFIRINOX for Borderline Resectable Pancreas Cancer: A New Treatment Paradigm? Oncologist (2014) 19(3):266–74. doi: 10.1634/theoncologist.2013-0273 PMC3958454 24569947

[11] Filippakopoulos P Qi J Picaud S Shen Y Smith WB Fedorov O. Selective Inhibition of BET Bromodomains. Nature (2010) 468(7327):1067–73. doi: 10.1038/nature09504 PMC3010259 20871596

[12] Shi J Vakoc CR. The Mechanisms Behind the Therapeutic Activity of BET Bromodomain Inhibition. Mol Cell (2014) 54(5):728–36. doi: 10.1016/j.molcel.2014.05.016 PMC4236231 24905006

[13] Doroshow DB Eder JP LoRusso PM. BET Inhibitors: A Novel Epigenetic Approach. Ann Oncol (2017) 28(8):1776–87. doi: 10.1093/annonc/mdx157 28838216

[14] Loven J Hoke HA Lin CY Lau A Orlando DA Vakoc CR. Selective Inhibition of Tumor Oncogenes by Disruption of Super-Enhancers. Cell (2013) 153(2):320–34. doi: 10.1016/j.cell.2013.03.036 PMC3760967 23582323

[15] Fehling SC Miller AL Garcia PL Vance RB Yoon KJ. The Combination of BET and PARP Inhibitors is Synergistic in Models of Cholangiocarcinoma. Cancer Lett (2020) 468:48–58. doi: 10.1016/j.canlet.2019.10.011 31605774 PMC7017643

[16] Karakashev S Zhu H Yokoyama Y Zhao B Fatkhutdinov N Kossenkov AV. BET Bromodomain Inhibition Synergizes With PARP Inhibitor in Epithelial Ovarian Cancer. Cell Rep (2017) 21(12):3398–405. doi: 10.1016/j.celrep.2017.11.095 PMC5745042 29262321

[17] Yang L Zhang Y Shan W Hu Z Yuan J Pi J. Repression of BET Activity Sensitizes Homologous Recombination-Proficient Cancers to PARP Inhibition. Sci Transl Med (2017) 9(400):eaal1645. doi: 10.1126/scitranslmed.aal1645 28747513 PMC5705017

[18] Miller AL Fehling SC Garcia PL Gamblin TL Council LN van Waardenburg R. The BET Inhibitor JQ1 Attenuates Double-Strand Break Repair and Sensitizes Models of Pancreatic Ductal Adenocarcinoma to PARP Inhibitors. EBioMedicine (2019) 44:419–30. doi: 10.1016/j.ebiom.2019.05.035 PMC6604668 31126889

[19] Al Bitar S Ballout F Monzer A Kanso M Saheb N Mukherji D. Thymoquinone Radiosensitizes Human Colorectal Cancer Cells in 2D and 3D Culture Models. Cancers (Basel) (2022) 14(6):1363. doi: 10.3390/cancers14061363 35326517 PMC8945905

[20] Franken NA Rodermond HM Stap J Haveman J van Bree C. Clonogenic Assay of Cells In Vitro. Nat Protoc (2006) 1(5):2315–9. doi: 10.1038/nprot.2006.339 17406473

[21] Zeng L Nikolaev A Xing C Della Manna DL Yang ES. CHK1/2 Inhibitor Prexasertib Suppresses NOTCH Signaling and Enhances Cytotoxicity of Cisplatin and Radiation in Head and Neck Squamous Cell Carcinoma. Mol Cancer Ther (2020) 19(6):1279–88. doi: 10.1158/1535-7163.MCT-19-0946 32371584

[22] Shorstova T Foulkes WD Witcher M. Achieving Clinical Success With BET Inhibitors as Anti-Cancer Agents. Br J Cancer (2021) 124(9):1478–90. doi: 10.1038/s41416-021-01321-0 PMC8076232 33723398

[23] Miller AL Garcia PL Fehling SC Gamblin TL Vance RB Council LN. The BET Inhibitor JQ1 Augments the Antitumor Efficacy of Gemcitabine in Preclinical Models of Pancreatic Cancer. Cancers (Basel) (2021) 13(14):3470. doi: 10.3390/cancers13143470 34298684 PMC8303731

[24] Cochran AG Conery AR Sims RJ 3rd. Bromodomains: A New Target Class for Drug Development. Nat Rev Drug Discovery (2019) 18(8):609–28. doi: 10.1038/s41573-019-0030-7 31273347

[25] Donati B Lorenzini E Ciarrocchi A. BRD4 and Cancer: Going Beyond Transcriptional Regulation. Mol Cancer (2018) 17(1):164. doi: 10.1186/s12943-018-0915-9 30466442 PMC6251205

[26] Baratta MG Schinzel AC Zwang Y Bandopadhayay P Bowman-Colin C Kutt J. An in-Tumor Genetic Screen Reveals That the BET Bromodomain Protein, BRD4, is a Potential Therapeutic Target in Ovarian Carcinoma. Proc Natl Acad Sci U S A (2015) 112(1):232–7. doi: 10.1073/pnas.1422165112 PMC4291641 25535366

[27] Feng Q Zhang Z Shea MJ Creighton CJ Coarfa C Hilsenbeck SG. An Epigenomic Approach to Therapy for Tamoxifen-Resistant Breast Cancer. Cell Res (2014) 24(7):809–19. doi: 10.1038/cr.2014.71 PMC4085766 24874954

[28] Garcia PL Miller AL Kreitzburg KM Council LN Gamblin TL Christein JD. The BET Bromodomain Inhibitor JQ1 Suppresses Growth of Pancreatic Ductal Adenocarcinoma in Patient-Derived Xenograft Models. Oncogene (2016) 35(7):833–45. doi: 10.1038/onc.2015.126 PMC6713275 25961927

[29] Lockwood WW Zejnullahu K Bradner JE Varmus H. Sensitivity of Human Lung Adenocarcinoma Cell Lines to Targeted Inhibition of BET Epigenetic Signaling Proteins. Proc Natl Acad Sci U S A (2012) 109(47):19408–13. doi: 10.1073/pnas.1216363109 PMC3511085 23129625

[30] Sahai V Kumar K Knab LM Chow CR Raza SS Bentrem DJ. BET Bromodomain Inhibitors Block Growth of Pancreatic Cancer Cells in Three-Dimensional Collagen. Mol Cancer Ther (2014) 13(7):1907–17. doi: 10.1158/1535-7163.MCT-13-0925 PMC4090266 24807963

[31] Engelke CG Parsels LA Qian Y Zhang Q Karnak D Robertson JR. Sensitization of Pancreatic Cancer to Chemoradiation by the Chk1 Inhibitor Mk8776. Clin Cancer Res (2013) 19(16):4412–21. doi: 10.1158/1078-0432.CCR-12-3748 PMC3745540 23804422

[32] Kausar T Schreiber JS Karnak D Parsels LA Parsels JD Davis MA. Sensitization of Pancreatic Cancers to Gemcitabine Chemoradiation by WEE1 Kinase Inhibition Depends on Homologous Recombination Repair. Neoplasia (2015) 17(10):757–66. doi: 10.1016/j.neo.2015.09.006 PMC4656803 26585231

[33] Maacke H Jost K Opitz S Miska S Yuan Y Hasselbach L. DNA Repair and Recombination Factor Rad51 is Over-Expressed in Human Pancreatic Adenocarcinoma. Oncogene (2000) 19(23):2791–5. doi: 10.1038/sj.onc.1203578 10851081

[34] Zhang X Ma N Yao W Li S Ren Z. RAD51 is a Potential Marker for Prognosis and Regulates Cell Proliferation in Pancreatic Cancer. Cancer Cell Int (2019) 19:356. doi: 10.1186/s12935-019-1077-6 31889908 PMC6935217

[35] Li X Baek G Ramanand SG Sharp A Gao Y Yuan W. BRD4 Promotes DNA Repair and Mediates the Formation of TMPRSS2-ERG Gene Rearrangements in Prostate Cancer. Cell Rep (2018) 22(3):796–808. doi: 10.1016/j.celrep.2017.12.078 29346775 PMC5843368

[36] Camero S Camicia L Marampon F Ceccarelli S Shukla R Mannarino O. BET Inhibition Therapy Counteracts Cancer Cell Survival, Clonogenic Potential and Radioresistance Mechanisms in Rhabdomyosarcoma Cells. Cancer Lett (2020) 479:71–88. doi: 10.1016/j.canlet.2020.03.011 32200036

[37] Wang J Wang Y Mei H Yin Z Geng Y Zhang T. The BET Bromodomain Inhibitor JQ1 Radiosensitizes non-Small Cell Lung Cancer Cells by Upregulating P21. Cancer Lett (2017) 391:141–51. doi: 10.1016/j.canlet.2017.01.031 28143717

[38] Wilson AJ Stubbs M Liu P Ruggeri B Khabele D. The BET Inhibitor INCB054329 Reduces Homologous Recombination Efficiency and Augments PARP Inhibitor Activity in Ovarian Cancer. Gynecol Oncol (2018) 149(3):575–84. doi: 10.1016/j.ygyno.2018.03.049 PMC5986599 29567272

[39] Mio C Gerratana L Bolis M Caponnetto F Zanello A Barbina M. BET Proteins Regulate Homologous Recombination-Mediated DNA Repair: BRCAness and Implications for Cancer Therapy. Int J Cancer (2019) 144(4):755–66. doi: 10.1002/ijc.31898 30259975

[40] He DD Shang XY Wang N Wang GX He KY Wang L. BRD4 Inhibition Induces Synthetic Lethality in ARID2-Deficient Hepatocellular Carcinoma by Increasing DNA Damage. Oncogene (2022) 41(10):1397–409. doi: 10.1038/s41388-022-02176-2 35017665

[41] Willett CG Czito BG Bendell JC Ryan DP. Locally Advanced Pancreatic Cancer. J Clin Oncol (2005) 23(20):4538–44. doi: 10.1200/JCO.2005.23.911 16002845

[42] Sharma RA Plummer R Stock JK Greenhalgh TA Ataman O Kelly S. Clinical Development of New Drug-Radiotherapy Combinations. Nat Rev Clin Oncol (2016) 13(10):627–42. doi: 10.1038/nrclinonc.2016.79 27245279

